# Efficacy of galcanezumab in migraine central sensitization

**DOI:** 10.1038/s41598-024-72282-6

**Published:** 2024-09-18

**Authors:** Daisuke Danno, Noboru Imai, Shigekazu Kitamura, Kumiko Ishizaki, Shoji Kikui, Takao Takeshima

**Affiliations:** 1 Headache Center, Department of Neurology, Tominaga Hospital, 1-4-48 Minatomachi, Naniwa Ward, Osaka, Japan; 2 Department of Neurology and Headache Center, Japanese Red Cross Shizuoka Hospital, Shizuoka, Japan; 3 Department of Neurology, Konan Kakogawa Hospital, Kakogawa, Hyogo Japan

**Keywords:** Migraine, Central sensitization, CGRP antibodies, Galcanezumab, Central effect, Interictal burden, Headache, Migraine

## Abstract

Galcanezumab, a monoclonal antibody targeting the calcitonin gene-related peptide pathway (CGRP mAb), acts peripherally due to its large size. However, recent studies have suggested that CGRP mAbs may also have a central mode of action. This study aimed to evaluate the central effects of galcanezumab on migraine central sensitization.This prospective real-world study was conducted at three headache centers in Japan between May 2021 and May 2022. Patients treated with galcanezumab for migraines were included in the study. The primary outcome was the change in the validated Central Sensitization Inventory (CSI) score from baseline to six months of treatment. We also assessed changes in the Allodynia Symptom Checklist (ASC-12) score. Eighty-six patients with migraine (73 female and 13 male) were analyzed. At 6 months, CSI and ASC-12 scores were significantly reduced compared to baseline (CSI: 36.0 vs. 29.3, *p* < 0.001; and ASC-12: 5.55 vs. 4.26, *p* < 0.01). Furthermore, these effects were observed as early as three months of treatment. In this study, we demonstrated the real-world efficacy of galcanezumab in improving central sensitization in migraine, with significant effects seen in the early phase of treatment.

*Trial registration*: This study was registered with UMIN-CTR on May 2, 2021 (UMIN000044096).

## Introduction

In the Global Burden of Disease Study 2021, the World Health Organization (WHO) ranked migraine as the first and second leading cause of global disability-adjusted life-years (DALYs) for people aged 5–19 years and 20–59 years respectively, indicating that the burden of migraine on patients' lives is very high^1^. Recently, the European Headache Federation updated the definition of resistant migraine as having failed at least 3 classes of migraine preventive medication and having at least eight debilitating headache days per month for at least three consecutive months without improvement, and refractory migraine as having failed all available preventive medication and having at least eight debilitating headache days per month for at least 6 consecutive months^2^. Patients with chronic migraine (CM) or medication overuse headache (MOH) are sometimes resistant or refractory to oral standard of care, and central sensitization is recognized as one of the explanations for non-response^3^. It has also been reported that central sensitization plays a critical role in the pathogenesis and development of chronic migraine^4^. Oral standard of care, including anti-seizure medications, tricyclic antidepressants, calcium channel blockers, and beta-blockers, or injection treatment with onabotulinumtoxinA (BoNT-A) have been used as prophylactic medications for patients with migraine. However, adherence, especially to oral standard of care at 12 months, was low at 17% to 20% due to side effects and/or lack of efficacy, which have emerged as clinical issues^5^. In addition, the study evaluating adherence to standard oral migraine prophylaxis reported that a high rate of treatment discontinuation was associated with increased healthcare resource use and costs for non-adherent patients^6^.

Recently, mAbs targeting the CGRP pathway have been developed as novel migraine-specific preventives^7^^.^ Galcanezumab and fremanezumab are monoclonal antibodies that target CGRP, whereas erenumab targets CGRP receptors. Galcanezumab was the first CGRP mAb to be launched in Japan in April 2021^8^^.^ The efficacy of galcanezumab has been demonstrated in phase 3 clinical trials not only in patients with episodic migraine (EM) but also in patients with CM and MOH^9–12^. Galcanezumab was also shown to be effective in patients with migraine in a real-world study in Japan^13^. In terms of the origin of migraine, not only peripheral but also central mechanisms are recognized to play important roles in the pathophysiology, and recruitment of brain areas such as the hypothalamus, pons, spinal trigeminal nucleus, thalamus, and visual and pain-processing cortical areas is thought to begin during the premonitory phase and contribute to the onset of pain and associated symptoms^14^^.^ Because CGRP mAbs have high molecular weights, they are believed to penetrate the blood–brain barrier (BBB) in very low amounts, and the effects of CGRP mAbs are thought to be exerted by targeting the peripheral part of the trigeminovascular system^15^. However, improvement or resolution of aura symptoms caused by the central phenomenon of cortical spreading depression (CSD) after treatment with galcanezumab or erenumab has been reported in two patients with migraine with aura^16^. In another study investigating the effects on central migraine symptoms in CGRP mAb responders, few patients reported recurrence of prodromal and associated symptoms without headaches. In this study, sleep changes and increased appetite and weight were also reported in responders, suggesting that CGRP mAbs may prevent central symptoms in patients with migraine^17^. In addition, a functional magnetic resonance imaging (fMRI) study of migraine patients treated with erenumab reported a significant reduction in activation in the hypothalamus only in responders, raising the possibility that CGRP mAbs have a direct central mode of action in migraine patients^18^. In another clinical trial investigating the effect of erenumab by examining resting-state functional connectivity changes in brain networks in patients with migraine using fMRI, it was also reported that patients receiving erenumab showed changes in functional connectivity within the cerebellar, thalamic, and periaqueductal gray matter networks compared with placebo, which were significantly associated with clinical improvement. The authors speculated that one of the possible mechanisms is that the small amount of mAbs that penetrate the BBB may exert an effect on the central nervous system (CNS)^19^. As mentioned above, previous reports have suggested that CGRP mAbs may have central effects; however, few reports have investigated the effects of CGRP mAbs on central sensitization. In this prospective study, we investigated the efficacy of galcanezumab in migraine central sensitization in a real-world setting using validated CSI, and evaluated the central effect of galcanezumab^20,21^.

## Methods

### Study design and participants

**Fig. 1:**
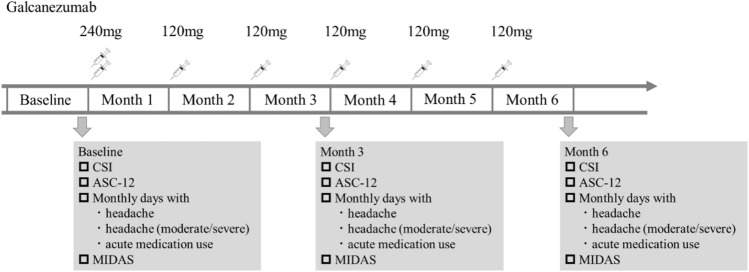
Study design. The 1-month baseline period was followed by a 6-month treatment period. Galcanezumab (240 mg) was administered during the treatment period, followed by monthly subcutaneous injections of galcanezumab (120 mg).

This was a prospective, multicenter, real-world study. We studied patients with episodic and chronic migraine who were diagnosed with migraine and had been treated at three headache centers in Japan (Tominaga Hospital Headache Center, Japanese Red Cross Shizuoka Hospital Headache Center, and Konan Hospital Headache Clinic) from May 2021 to May 2022 by at least one of the following headache specialists: TT, DD, NI, ShiK, KI, and ShoK. The inclusion criteria were patients aged 20–65 years with a diagnosis of migraine who were treated with galcanezumab as part of their usual treatment by their health insurance (1.1 migraine without aura [MO], 1.2 migraine with aura [MA], and 1.3 CM) as defined by the International Classification of Headache Disorders, 3rd edition [ICHD-3])^22^. Patients with secondary headaches other than MOH were excluded from the study. Patients were also excluded if they had trigeminal autonomic cephalgia (TACs) or new daily persistent headache (NDPH). The 1-month baseline period was followed by the 6-month treatment period. During the treatment period, a loading dose of galcanezumab (240 mg) was administered, followed by monthly subcutaneous injections of galcanezumab (120 mg). The regimen of concomitant prophylactic medication had to be stable 2 months prior to baseline and remained stable from baseline until 6 months of galcanezumab treatment. We used a structured self-report questionnaire to collect data. These data were reviewed face-to-face with the patients in the clinic (Fig. 1).

### Collected variables and outcomes

The primary outcome was the change in the CSI score from baseline to 6 months after treatment with galcanezumab. CSI is a self-report screening tool used to assess the severity of symptoms in central sensitivity syndrome (CSS). CSS shares common features, including pain, fatigue, insomnia, anxiety, and depression, suggesting that they may, at least in part, share a common underlying etiology of central sensitisation^20^. The CSI consists of 25 items asking about health-related symptoms related to central sensitization, and each item rates the severity of the symptoms on a 5-point Likert scale (0 = never, 1 = rarely, 2 = sometimes, 3 = often, and 4 = always). In a study to establish relevant severity levels for CSI, 167 patients with CSSs including 58 patients with migraine/tension-type headache were analyzed. The total CSI scores were categorized according to severity levels as follows: 0–29, subclinical; 30–39, mild; 40–49, moderate; 50–59, severe; and 60–100, extreme,^20,21,23^ (Supplementary Table 1). In this study, we used the validated Japanese version of the CSI^21^.

The secondary outcomes were the change from baseline in the CSI score at 3 months, ASC-12, monthly headache days, monthly days with headache of at least moderate severity, monthly days with acute medication use, and Migraine Disability Assessment (MIDAS) at 3 and 6 months (Fig. 1).

### Statistical analysis

All clinical data were collected using Microsoft Excel (Microsoft Corporation, Redmond, WA, USA). Friedman's test with Bonferroni correction was used to assess the changes from baseline for the MIDAS at months 3 and 6. A multiple linear regression analysis was used to identify causal relationships between CSI scores and other parameters. Repeated measures ANOVA with Bonferroni correction was used to assess the remaining items. All analyses were primary analyses of data and were performed with a two-tailed threshold *p*-value of 0.05, using the EZR software program^24^. The sample size was based on available data, and statistical power was not calculated prior to the study.

## Results

### Disposition and baseline characteristics of the patients

**Fig. 2:**
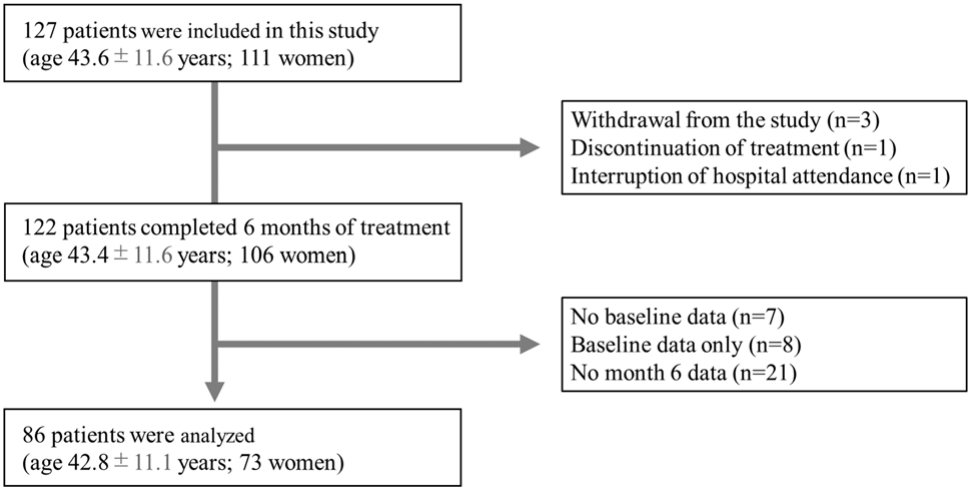
Study flowchart.

One hundred twenty-seven patients with migraine (female, n = 111; male, n = 16; mean age at baseline, 43.6 years [range, 20–65 years]) were enrolled. Subsequently, three patients withdrew from the study after obtaining their consent. One patient discontinued treatment owing to lack of efficacy, and one patient discontinued clinic visits. One hundred twenty-two patients with migraine (female, n = 106; male, n = 16; mean age at baseline, 43.4 years [range: 20–65 years]) completed 6 months of treatment; however, 7 patients did not provide a structured self-report questionnaire on parameters including headache status at baseline. Twenty-one patients did not provide it at the 6-month follow-up examination. Eight patients only provided their baseline data. In addition, 6 patients did not provide their 3-month follow-up data; however, we were able to obtain their 6-month follow-up data, which was the primary outcome. Therefore these 6 cases were included in the analysis. Finally, we analyzed the data from 86 patients (female, n = 73; male, n = 13; mean age at baseline, 42.8 years [range: 20–64 years]) (Fig. 2).

The diagnoses were as follows: EM (n = 26, 30.2%), CM with MOH (n = 40, 46.5%), and CM without MOH (n = 20, 23.3%). There were 3 patients with MA in CM with MOH and 1 patient with MA in CM without MOH. The mean number of days per month with moderate and severe headache at baseline was 7.7 and 3.6, respectively. A stable dose of concomitant standard oral preventive medication was prescribed to 59 patients (68.6%) during the study. The details of the baseline patient demographics are shown in the table (Table 1).

**Table 1:** Patient demographics (n = 86).

Age and sex		
Age at study [years (mean ± SD)]		42.8 ± 11.1
Age at onset of migraine [years (mean ± SD)] *n = 79		15.5 ± 7.3
Females [cases (%)]		73 (84.9%)
Diagnosis		
EM [cases (%)]		26 (30.2%)
CM with MOH [cases (%)]		40 (46.5%)
CM without MOH [cases (%)]		20 (23.3%)
Characteristics of headache		
Attack duration [hours, median (IQR)]		6 (4.8–13.3)
Headache laterality [cases (%)]	Strictly unilateral	6 (7.0%)
	Unilateral side variable	15 (17.4%)
	Bilateral or unilateral	50 (58.1%)
	Always bilateral	15 (17.4%)
Monthly days with moderate headaches (mean ± SD)		7.7 ± 6.1
Monthly days with severe headaches (mean ± SD)		3.6 ± 4.8
Pulsating quality [cases (%)]		41 (47.7%)
Aggravation by routine physical activity [cases (%)]		66 (76.7%)
Nausea and/or vomiting [cases (%)]		55 (64.0%)
Photophobia [cases (%)]		71 (82.6%)
Phonophobia [cases (%)]		60 (69.8%)
Osmophobia [cases (%)]		55 (64.0%)
Treatments		
Days with acute medication use/month (mean ± SD)		10.4 ± 6.7
Concomitant preventive treatment [cases (%)]		59 (68.6%)
Past medical history		
Restless leg syndrome [cases (%)]		2 (2.3%)
Temporomandibular joint disorder [cases (%)]		13 (15.1%)
Irritable bowel syndrome [cases (%)]		11 (12.8%)
Multiple chemical sensitivities [cases (%)]		2 (2.3%)
Neck injury (including whiplash) [cases (%)]		12 (14.0%)
Anxiety or panic attacks [cases (%)]		14 (16.3%)
Depression [cases (%)]		18 (20.9%)

SD, standard deviation; EM, episodic migraine; CM, chronic migraine; MOH, medication overuse headache; IQR, interquartile range.

* Seven patients could not recall their age at onset.

### Efficacy of galcanezumab in central sensitization

**Fig. 3:**
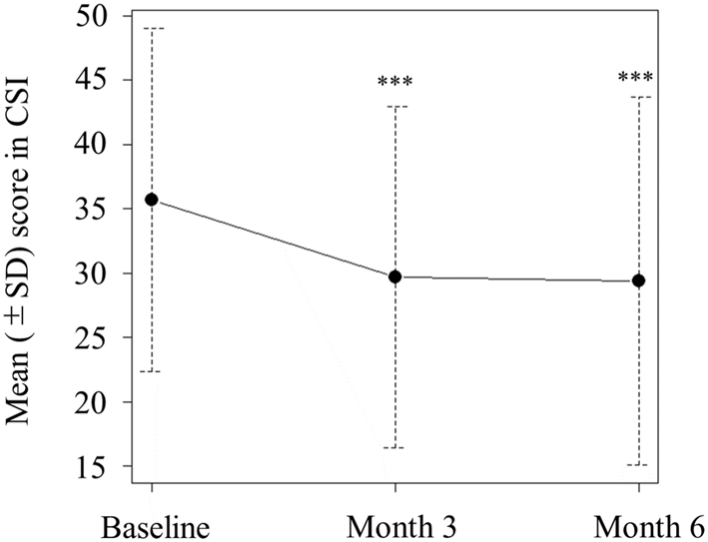
Overall CSI score changes. The mean CSI score at baseline was 36.0 and showed a significant decrease to 29.7 at 3 months (*p* < 0.001 [7e−8] vs. baseline) and to 29.3 at 6 months (*p* < 0.001 [1e−8] vs. baseline). CSI: Central Sensitization Inventory; SD: standard deviation.

**Fig. 4:**
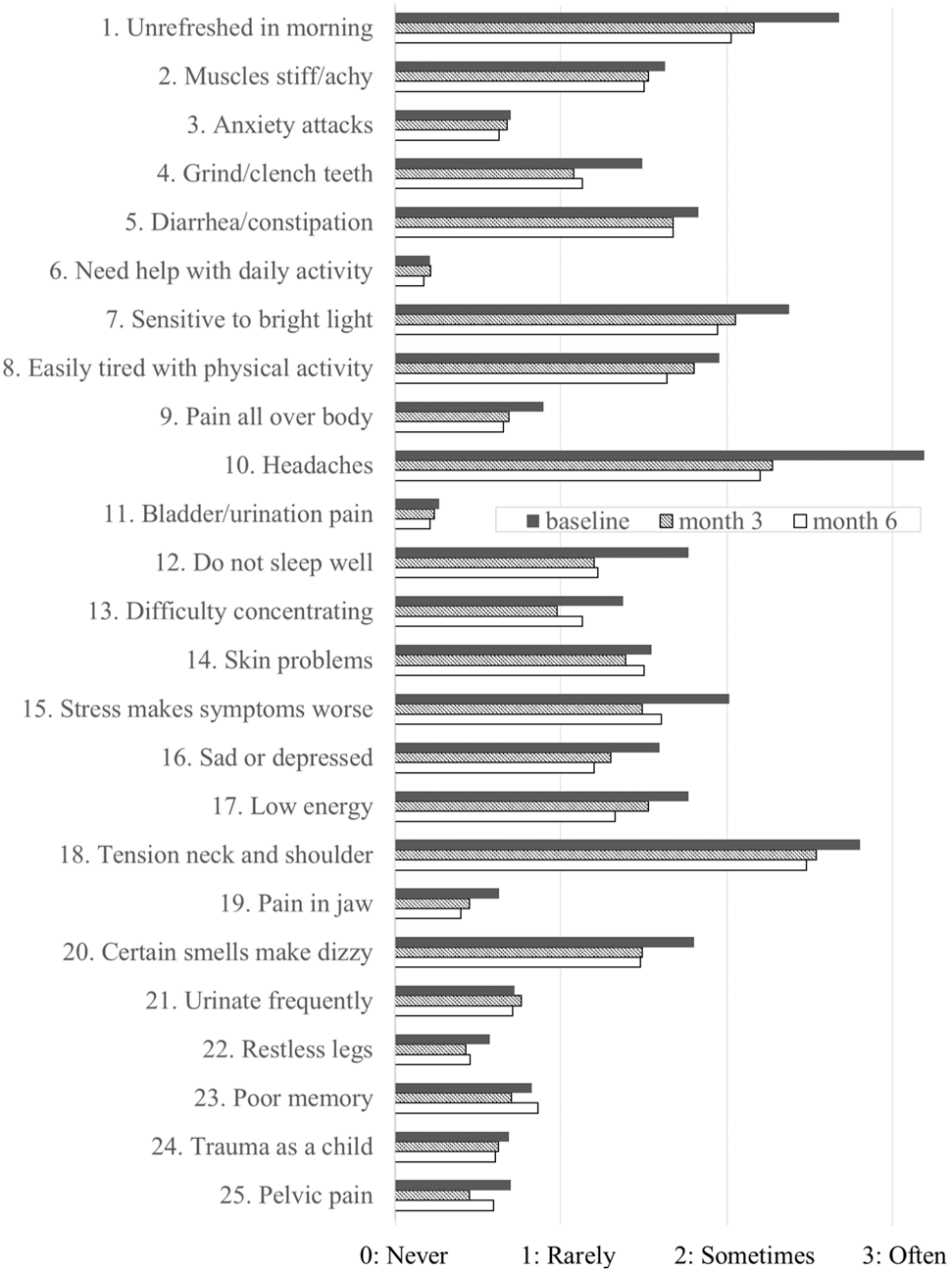
The breakdown of the 25 items in the CSI. At 6 months, the scores for the majority of items improved in comparison to baseline. CSI: Central Sensitization Inventory.

The mean CSI score at baseline was 36.0 (range, 9–75) and decreased significantly to 29.3 (range, 5–83) at 6 months (*p* < 0.001 vs. baseline). For the majority of the items, the CSI scores improved at 6 months compared to baseline. Furthermore, the score also decreased significantly to 29.7 (range, 2–78) at 3 months (*p* < 0.001 vs. baseline), which revealed that galcanezumab improves the severity of symptoms related to central sensitization in migraine at an early stage after the initiation of treatment (Figs. 3,4).

In addition, we also compared the mean 24-item CSI scores, omitting one item asking if patients had headaches, because the direct effect of galcanezumab on headaches could affect the overall CSI score. We found that the mean 24-item CSI score also decreased significantly (baseline: 32.8 vs. 6 months:27.1, *p* < 0.001).

The mean ASC-12 score, which reflects the degree of skin allodynia, also showed a significant reduction after 3 months (baseline: 5.55 vs. 3 months:4.09, *p* < 0.01). Cutaneous allodynia is a representative marker of central sensitization; therefore, the results also suggest that galcanezumab improves central sensitization in migraine (Table 2).

**Table 2:** Changes in allodynia and headache (n = 86).

		Mean ± SD	*p* value (vs. baseline)
ASC-12 score	Baseline	5.55 ± 5.47	
	Month 3	4.09 ± 4.97	0.0060^††^
	Month 6	4.26 ± 5.05	0.0092^††^
Headache days/month	Baseline	17.1 ± 8.6	
	Month 3	10.8 ± 9.8	0.0000^†††^ (5e−08)
	Month 6	10.7 ± 9.1	0.0000^†††^ (3e−11)
Headache days of at least moderate severity/month	Baseline	11.3 ± 8.1	
	Month 3	6.0 ± 7.3	0.0000^†††^ (2e−11)
	Month 6	5.3 ± 6.8	0.0000^†††^ (3e−11)
Days with acute medication use/month	Baseline	10.4 ± 6.7	
	Month 3	7.3 ± 6.1	0.0003^†††^
	Month 6	7.5 ± 6.9	0.0000^†††^ (1e−5)

ASC-12: Allodynia Symptom Checklist, SD: standard deviation.

^††^*p* < 0.01, ^†††^*p* < 0.001.

### Headache, acute medication utilization, and disability

The mean number of monthly headache days and the number of monthly headache days of at least moderate severity were 17.1 and 11.3, respectively, at baseline. These were significantly reduced from 3 months (10.8 and 6.0, respectively; *p* < 0.001 vs. baseline), and efficacy was maintained until 6 months. With the improvement in headache, the number of monthly days with acute medication use also decreased from 10.4 at baseline through months 3 and 6 (7.3 and 7.5, respectively; *p* < 0.001 vs. baseline) (Table 2).

**Fig. 5:**
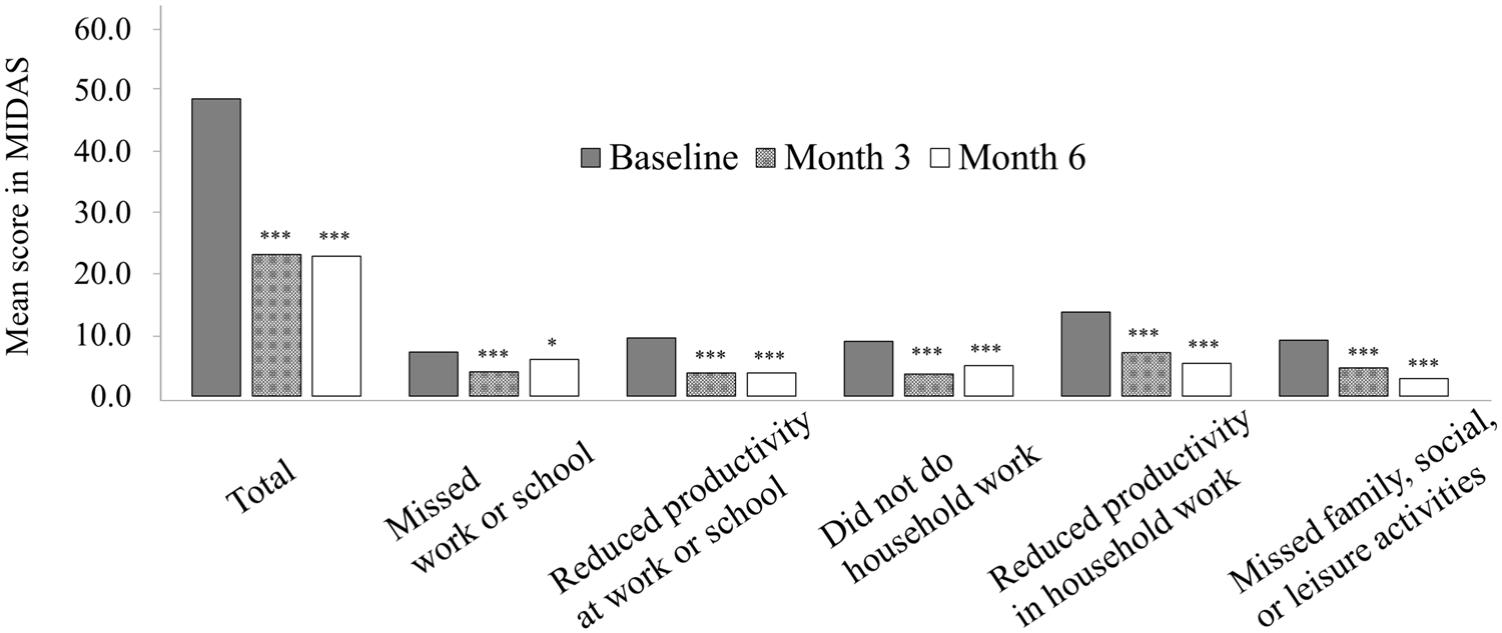
The mean MIDAS score changes. The mean total MIDAS score improved significantly from 48.6 at baseline to 23.1 at 3 months (*p* < 0.001 vs. baseline) and 22.8 at 6 months (*p* < 0.001 vs. baseline). Each of the MIDAS items also showed significant improvement in comparison to baseline from 3 to 6 months. MIDAS: Migraine Disability Assessment.

In terms of disability caused by migraine, the mean total MIDAS score improved significantly from 48.6 at baseline to 22.8 at six months (*p* < 0.001 vs. baseline). Each of the MIDAS items also showed significant improvement from 3 to 6 months in comparison to baseline, including days missed from work or school, days missed from household work, and days missed from social or leisure activities (Fig. 5).

### Causal relationships between decrease of CSI score and other parameters

**Fig. 6:**
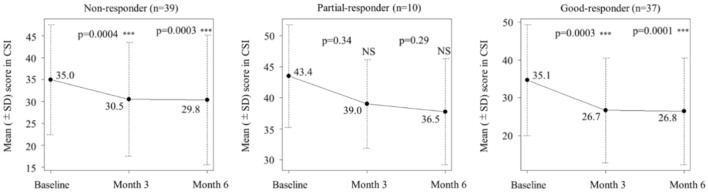
CSI score changes categorized by the responder rate. The mean CSI scores were significantly decreased (from baseline) at 3 and 6 months in the non-responders (responder rate < 30%) and good responders (responder rate ≥ 50%). CSI: Central Sensitization Inventory; NS: not significant.

A multiple linear regression analysis of factors predicting CSI score reduction was performed to investigate whether the positive effect on central sensitization was correlated with improvements in other parameters, and the results showed that only ASC-12 score reduction was significantly associated with CSI score reduction (*p* = 0.0312). In contrast, other explanatory variables, including reductions in monthly headache days (*p* = 0.3672), monthly days with at least moderate headache (*p* = 0.0612), acute medication use (*p* = 0.2286), and the MIDAS score (*p* = 0.6144), did not significantly predict CSI score reduction at 6 months (Table 3). We also analyzed the reduction in CSI score for non-responders (responder rate < 30%; n = 39), partial responders (responder rate 30–49%; n = 10), and good responders (responder rate ≥ 50%; n = 37) and found that the CSI score was significantly reduced even in the non-responder group (baseline: 35.0 vs. 6 months:29.8, *p* = 0.0003) (Fig. 6).

**Table 3:** Multiple linear regression analysis for the prediction of CSI score reduction at 6 months (n = 86).

Variables	Regression coefficient estimate	CI 95%	*p* value
ASC-12 score reduction	0.531	0.049; 1.012	0.0312^†^
Headache days/month reduction	0.150	− 0.179; 0.478	0.3672
Headache days of at least moderate severity/month reduction	0.343	− 0.016; 0.702	0.0612
Days with acute medication use/month reduction	− 0.252	− 0.666; 0.162	0.2286
MIDAS score reduction	0.012	− 0.034; 0.058	0.6144

^†^*p* < 0.05.

CSI: Central Sensitization Inventory.

ASC-12: Allodynia Symptom Checklist, CI: Confidence of Interval.

MIDAS: Migraine Disability Assessment.

### Safety and tolerability

Three patients reported injection site reactions; however, no serious adverse events were reported, and no patient discontinued treatment owing to adverse events during the study.

## Discussion

### CSI

Central sensitization is a physiological phenomenon in which the CNS becomes hypersensitive to both noxious and non-noxious stimuli^25^. Quantitative sensory testing (QST), including static and dynamic psychophysical tests to quantify somatosensory function in response, has been utilized as a method for measuring central sensitization; however, its clinical applicability is limited because it is labor intensive^21^. CSI is a validated screening tool used to assess the symptoms associated with central sensitization in CSSs, and statistically significant CSI-QST correlations for all 5 QST modalities, including conditioned pain modulation, temporal summation, pressure pain threshold, heat pain threshold, and cold pain threshold, have been reported in a meta-analysis including 33 studies with 3314 subjects^20,26^. Nearly 80% of patients with migraine have been reported to experience allodynia, and migraine is recognized as one of the main disorders associated with central sensitization^27^. In a study to establish a cutoff score for CSI, 89 patients with CSSs were included and 47 of them had migraine/tension-type headache with headache being the most common diagnosis. A receiver operating characteristic (ROC) analysis showed that a cutoff score of 40 discriminated patients with CSS from controls with 81% sensitivity and 75% specificity^25^.

Several studies have assessed the symptoms related to central sensitization in migraine using CSI. In a study using CSI for patients with migraine diagnosed by headache specialists according to the ICHD-3 (beta version), patients with migraine were 3 times more likely to have central sensitization than healthy subjects^28–30^. These findings suggest that CSI is a practical tool for assessing the severity of symptoms related to central sensitization in migraine.

### Efficacy of galcanezumab for central sensitization

In this prospective study, we showed that galcanezumab significantly reduced the severity of symptoms related to central sensitization in patients with migraine after six months of treatment. The main area where galcanezumab exerts its effects is thought to be the peripheral nervous system owing to its large size. However, several studies have suggested that CGRP mAbs may also act on CNS^16–18^. Recently, we also reported a case series of patients with hemiplegic migraine in whom the motor aura improved after treatment with galcanezumab, even though the pathophysiology of migraine aura, including motor weakness, has been recognized as the result of CSD, an electrophysiological activity in the CNS^7^. While the exact mechanism of action of galcanezumab in the CNS is unclear, two hypotheses can be considered. The first hypothesis was that this is a secondary effect. Repeated activation of trigeminovascular neurons, that is, repeated activation of pain pathways, eventually leads to CNS dysfunction, resulting in central sensitisation^31^. It has been reported that the 75% response to CGRP mAb treatment was positively associated with allodynia and unilateral pain, and the authors suggested that CGRP contributes to the sensitization of second-order nociceptive neurons, which favors the development of central sensitization^32^. In another study investigating whether 3 months of treatment with galcanezumab alters various migraine symptoms, the incidence of aura followed by headache was reported to be reduced in responders, non-responders, and super-responders but not in super non-responders^33^. It is also reported in the study for 12 patients with either migraine with or without aura, that CGRP mAbs significantly reduced the mean number of migraine days without aura, but did not affect the frequency of migraine attacks with aura. Whereas, CGRP mAbs reduced both the intensity and duration of the headache phases of migraine with aura. The authors of the study speculated that CGRP mAbs do not affect neuronal and vascular events related to CSD and that conversely, CGRP mAbs may be able to counteract CSD-induced sensitization of the trigemino-vascular pathway^34^. Furthermore, in the study of 572 migraine patients treated with CGRP mAbs, ultra-late responders (i.e., patients who achieved a ≥ 50% response after > 24 weeks) accounted for 15.7% of patients (90/572), and the authors of the study speculated that CGRP mAbs act centripetally i.e., first desensitizing peripheral trigeminal nociceptors and then reversing central sensitization^35^. In view of these findings, we speculate that blockade of the peripheral CGRP pathway and reduction in the number of pain/nociceptive signals may conversely inhibit CNS hyperactivity, leading to the amelioration of central sensitization. The second hypothesis is that the mechanism involves a direct effect of galcanezumab on the CNS. Interestingly, an animal study investigating the distribution of galcanezumab in the peripheral nervous system and CNS using iodine-125 reported that although the central levels of galcanezumab were low (< 0.4% of plasma), the authors concluded that the central effect of galcanezumab could not be excluded^36^. CGRP is known to be expressed not only in the trigeminal vascular system but also in the CNS, and in general, CGRP is abundantly expressed in the gray matter and neurons^37^. In a study investigating whether the electrophysiological effects of peripherally acting erenumab occur centrally, erenumab reduced the area under the curve of the nociceptive blink reflex after 1 month of treatment, suggesting that erenumab may have central effects earlier in the brainstem and a possible direct effect on the central nervous system^38^. Furthermore, in a prospective longitudinal study using high-resolution MRI to investigate the effect of peripherally acting galcanezumab on brain morphometry in patients with migraine, a reduction in cortical thickness in comparison to baseline was observed in responders after three months of treatment, suggesting that the reduction in headache may lead to changes that reflect the recovery process from maladaptive neuronal activity. The authors in the study interpreted this finding as "site of action" of galcanezumab is peripheral, and its prophylactic "mechanism of action" is central^39^. In our study, ASC-12 score reduction was the only factor significantly associated with CSI score reduction at 6 months in the multiple linear regression analysis, and improvement in headache days did not predict improvement in CSI. Furthermore, a significant CSI score reduction was observed even in the non-responder group. From these results, we speculated that galcanezumab improved central sensitization in migraine and that the improvement was not only due to a secondary effect after headache relief with galcanezumab but also a possible direct effect.

The true mechanism of action of galcanezumab in reducing the severity of symptoms associated with central sensitization in migraine is not clear at present and needs to be elucidated in the future.

### Early improvement of central sensitization

In our study, we have shown that galcanezumab improves migraine central sensitization from an early stage after three months of treatment. In a study using CSI to assess central sensitization in patients with CSS, the CSI score of patients with CSS was significantly higher than that of healthy controls, and the mean CSI score of 5,188 healthy subjects was reported to be 15.8^40^. In our results, the CSI score improved significantly from 36.0 at baseline to 29.7 at 3 months and 29.3 at 6 months; however, the scores were still higher than those of healthy subjects. Our interpretation of these findings is that we are only looking at the initial stage of improvement in migraine central sensitization with galcanezumab treatment. Therefore, future studies with long-term treatment will deepen our understanding of the mechanism of action of galcanezumab in migraine central sensitization.

### Improvement in disability

In our study, we observed a significant improvement in MIDAS score after galcanezumab treatment. Improving disability in daily life is one of the most important goals in the treatment of migraine because the patient's burden is not only the headache burden during attacks but also the so-called interictal burden between attacks. Interictal burden includes different types of components, consisting of both emotional and non-emotional symptoms, such as anxiety/depressive symptoms, and visual perceptual changes/hypersensitivity. We tended to focus more on the ictal burden and neglect the interictal burden; however, recently, the interictal burden has been recognized as critical and has received increased attention in migraine practice^41,42^. Interestingly, on the other hand, the CSI consists of items about a variety of symptoms that are also seen during the interictal period, such as asking if there is difficulty concentrating, feeling sad, sensitivity to light, osmophobia, insomnia, etc.^20^. This means that there may be an overlap between the various symptoms observed in migraine patients with central sensitization and those observed during the interictal period. In the present study, galcanezumab reduced CSI and allodynia scores, indicating an improvement in the central sensitization of migraine. This suggests that galcanezumab may have improved not only the headache burden, but also the interictal burden as a result of the improvement in central sensitization, which may have contributed to the improvement in the MIDAS score. In fact, a study using the four-item Migraine Interictal Burden Scale (MIBS-4) to assess the burden between migraine attacks reported that galcanezumab resulted in a significant reduction in interictal burden^42^. However, the relationship between central sensitization and interictal burden in migraine patients requires further clarification.

### Strengths and limitations

The strength of this study lies in its prospective demonstration of the efficacy of peripherally acting galcanezumab in improving central sensitization in migraine, as measured using CSI in a real-world setting.

However, the present study has some limitations. First, it is crucial to mention the method used to assess central sensitization. CSI is not a tool to directly assess the severity of central sensitization but to assess the severity of symptoms associated with central sensitisation^20^. The International Association for the Study of Pain (IASP) defines central sensitization as “increased responsiveness of nociceptive neurons in the central nervous system to their normal or subthreshold afferent input”^43,44^. In addition to this traditional "bottom-up" model defined by the IASP, a "top-down" model of central sensitization, that is, an augmented pain process in the CNS which is maintained independently of nociceptive input, has also been proposed^45^; however, the problem is that there is currently no structured definition of the term “central sensitization” based on expert consensus^43^. Central sensitization has been demonstrated in animals using direct electrophysiological recordings from central neurons, which cannot be conducted in humans^43^. A recent meta-analysis found statistically significant correlations between total CSI scores and QST modalities, with the strongest associations identified between CSI scores and pain threshold testing^26^. Because QST involves a series of standardized tests that allow a degree of objectivity in the measurement and quantification of results, it may be tempting to conclude that QST methods are superior to patient self-reporting of symptoms for identifying central sensitization. However, even with QST, some inconsistencies have been reported due to different patient instructions, cultural differences, age, or different criteria for allodynia^26,46^. In addition, both QST and CSI can detect signs of central sensitization, but neither provides enough information to determine whether patients have central sensitisation^26^. Similarly, ASC-12 is not a tool to detect central sensitization, but to determine the level of allodynia symptoms during headache attacks^47,48^. In the study in which 22 female patients with CM were evaluated for 3 months after single injections of BoNT-A, the ASC-12 score significantly decreased with BoNT-A injection, on the other hand, no significant change was observed in thermal thresholds measured by QST. The authors of the study speculated that the improvement in allodynia indicates that BoNT-A reduces central sensitisation^47^; however, the discrepancy between ASC-12 and QST results suggests difficulty in assessing central sensitization. As mentioned above, it has not been possible to directly measure central sensitization in humans, nor have there been any methods or tools to precisely measure central sensitization^26,40^. Indeed, in our study, it was not possible to measure the severity of central sensitization by measuring CSI or ASC-12, and it cannot be completely excluded that the improvement in these scores was a secondary effect of headache improvement. However, patients with disorders associated with central sensitization are reported to have associated symptoms, including sleep disturbance, brain fog, and sensitivity to environmental stimuli, which are included in the CSI^20^. It is not possible to measure central sensitization directly or precisely, and one of the proposed solutions is that a variety of different measures within a test battery, possibly including the QST and CSI, may provide the best information in a clinical settin^26,43^. We did not measure QST, but combined two scores to measure the symptoms related to central sensitization, including CSI and ASC-12. In the present study, after 6 months of treatment with galcanezumab, (i) both CSI and ASC-12 improved significantly, (ii) only ASC-12 improvement predicted CSI improvement in multiple linear regression analysis, and (iii) CSI improved even in non-responders, suggesting that there has been an improvement in central sensitization with galcanezumab. Although central sensitization is an important topic in the field of migraine, it is not easy to assess central sensitization in daily clinical practice. CSI is a self-report screening tool that does not require complicated measurement tasks^20,21^. The strength of CSI is that it can be used to assess the overall status of central sensitization, (i.e., the overall allodynic load at the time of assessment); therefore, we believe that CSI is currently a practical tool to assess central sensitization in migraine. Development of migraine-specific central sensitization assessment tools will be of interest in the future.

Second, regarding the study design, we did not have a control group and did not investigate the effect of switching between CGRP mAbs. The placebo effect is a well-recognized central effect, and we cannot exclude the possibility that galcanezumab is completely unrelated to the central events that were observed; therefore, a prospective randomized controlled trial is warranted in the future. In terms of switching effects, switching between classes of anti-CGRP mAb and anti-CGRP receptor mAb has been reported to be potentially effective in a relevant proportion of patients with migraine who did not respond to the first CGRP mAb^49,50^; no differences were reported between patients who switched from anti-CGRP mAb to anti-CGRP receptor mAb or vice versa in the cohort selected by 3 variables (i.e., MIDAS score, monthly headache days, and monthly analgesic days)^50^. On the other hand, it has been also suggested that differences between mAb classes targeting the same pathways may confer differential efficacy^49^. Additionally, an fMRI study reported that galcanezumab specifically reduced hypothalamic activation in both responders and non-responders, whereas erenumab did not reduce hypothalamic activation in non-responders. Instead, erenumab specifically reduces activation in the operculum, insula, thalamus, and cerebellum, and there may be differences in the effects of mAb classes^18,51,52^. Although our study only examined galcanezumab, which was on the market in Japan at the start of our trial, future studies are expected to clarify whether switching has an effect on central sensitization in migraine.

## Conclusions

In this prospective study, we demonstrated the efficacy of galcanezumab in improving symptoms related to central sensitization in migraine patients. Galcanezumab, which acts peripherally, showed a significant reduction in the CSI and ASC-12 scores as early as 3 months, and an early effect of galcanezumab on central sensitization was revealed. Although it remains inconclusive and controversial whether galcanezumab acts directly in the CNS, our study demonstrated its real-world efficacy in improving central sensitization in migraine. A future study investigating the long-term efficacy for central sensitization in migraine is warranted, as early efficacy may be the initial stage of improvement.

## Supplementary Information

Supplementary Information 1.

## Data Availability

The datasets used in this study are available from the corresponding author upon reasonable request.

## Abbreviations

ASC-12: Allodynia Symptom Checklist
BBB: Blood–brain barrier
BoNT-A: OnabotulinumtoxinA
CI: Confidence interval
CGRP: Calcitonin gene-related peptide
CM: Chronic migraine
CNS: Central nervous system
CSD: Cortical spreading depression
CSI: Central Sensitization Inventory
CSS: Central sensitization syndrome
DALY: Disability-adjusted life-years year
EM: Episodic migraine
fMRI: Functional magnetic resonance imaging
IASP: International Association for the Study of Pain
ICHD-3: International Classification of Headache Disorders, third edition
IQR: Interquartile range
MA: Migraine with aura
mAb: Monoclonal antibody
MIBS-4: Four-item Migraine Interictal Burden Scale
MIDAS: Migraine Disability Assessment
MO: Migraine without aura
MOH: Medication-overuse headache
NDPH: New daily persistent headache
NS: Not significant
QST: Quantitative sensory testing
ROC: Receiver operating characteristic
SD: Standard deviation
TAC: Trigeminal autonomic cephalgia
WHO: World Health Organization
