# Supplementary material for: Efficacy of galcanezumab in migraine central sensitization
Source: Sci Rep. 2024 Sep 18;14:21824. doi: 10.1038/s41598-024-72282-6 (PMC11410828; doi:10.1038/s41598-024-72282-6)
Supplement: Supplementary file 1 — Supplementary Information 1. [file 41598_2024_72282_MOESM1_ESM.docx]

**Supplementary material**

**Table 1: Central Sensitization Inventory**

| Please circle the best response to the right of each statement. | | | | | | |
| --- | --- | --- | --- | --- | --- | --- |
| 1 | I feel unrefreshed when I wake up in the morning. | Never | Rarely | Sometimes | Often | Always |
| 2 | My muscles feel stiff and achy. |  |  |  |  |  |
| 3 | I have anxiety attacks. |  |  |  |  |  |
| 4 | I grind or clench my teeth. |  |  |  |  |  |
| 5 | I have problems with diarrhea and/or constipation. |  |  |  |  |  |
| 6 | I need help in performing my daily activities. |  |  |  |  |  |
| 7 | I am sensitive to bright lights. |  |  |  |  |  |
| 8 | I get tired very easily when I am physically active. |  |  |  |  |  |
| 9 | I feel pain all over my body. |  |  |  |  |  |
| 10 | I have headaches. |  |  |  |  |  |
| 11 | I feel discomfort in my bladder and/or burning when I urinate. |  |  |  |  |  |
| 12 | I do not sleep well. |  |  |  |  |  |
| 13 | I have difficulty concentrating. |  |  |  |  |  |
| 14 | I have skin problems such as dryness, itchiness or rashes. |  |  |  |  |  |
| 15 | Stress makes my physical symptoms get worse. |  |  |  |  |  |
| 16 | I feel sad or depressed. |  |  |  |  |  |
| 17 | I have low energy. |  |  |  |  |  |
| 18 | I have muscle tension in my neck and shoulders. |  |  |  |  |  |
| 19 | I have pain in my jaw. |  |  |  |  |  |
| 20 | Certain smells, such as perfumes, make me feel dizzy and nauseated. |  |  |  |  |  |
| 21 | I have to urinate frequently. |  |  |  |  |  |
| 22 | My legs feel uncomfortable and restless when I am trying to go to sleep at night. |  |  |  |  |  |
| 23 | I have difficulty remembering things. |  |  |  |  |  |
| 24 | I suffered trauma as a child. |  |  |  |  |  |
| 25 | I have pain in my pelvic area. |  |  |  |  |  |
|  | | | | | Total　= |  |

The CSI consists of 25 items asking about health-related symptoms, and each item rates the severity of the symptoms on a 5-point Likert scale (0=never, 1=rarely, 2=sometimes, 3=often, and 4=always). The total scores were categorized according to the degree of CSI severity as follows: 0–29, subclinical; 30–39, mild; 40–49, moderate; 50–59, severe; and 60–100, extreme^20, 23)^.

CSI: Central Sensitization Inventory
